# Metapopulation Model from Pathogen’s Perspective: A Versatile Framework to Quantify Pathogen Transfer and Circulation between Environment and Hosts

**DOI:** 10.1038/s41598-018-37938-0

**Published:** 2019-02-08

**Authors:** Shi Chen, Cristina Lanzas, Chihoon Lee, Gabriel L. Zenarosa, Ahmed A. Arif, Michael Dulin

**Affiliations:** 1 Department of Public Health Sciences, University of North Carolina Charlotte, Charlotte, North Carolina 28223 USA; 2 Department of Population Health and Pathobiology, North Carolina State University, Raleigh, North Carolina 27607 USA; 3 School of Business, Stevens Institute of Technology, Hoboken, New Jersey 07030 USA; 4 Department of Systems Engineering and Engineering Management, University of North Carolina Charlotte, Charlotte, North Carolina 28223 USA

## Abstract

Metapopulation models have been primarily explored in infectious disease epidemiology to study host subpopulation movements and between-host contact structures. They also have the potential to investigate environmental pathogen transferring. In this study, we demonstrate that metapopulation models serve as an ideal modeling framework to characterize and quantify pathogen transfer between environment and hosts. It therefore unifies host, pathogen, and environment, collectively known as the epidemiological triad, a fundamental concept in epidemiology. We develop a customizable and generalized pathogen-transferring model where pathogens dwell in and transferring (via contact) between environment and hosts. We analyze three specific case studies: pure pathogen transferring without pathogen demography, source-sink dynamics, and pathogen control via external disinfection. We demonstrate how pathogens circulate in the system between environment and hosts, as well as evaluate different controlling efforts for healthcare-associated infections (HAIs). For pure pathogen transferring, system equilibria can be derived analytically to explicitly quantify long-term pathogen distribution in the system. For source-sink dynamics and pathogen control via disinfection, we demonstrate that complete eradication of pathogens can be achieved, but the rates of converging to system equilibria differ based on specific model parameterization. Direct host-host pathogen transferring and within-host dynamics can be future directions of this modeling framework by adding specific modules.

## Introduction

Mathematical models are powerful tools to study infectious disease dynamics, including the commonly used compartment models, particularly host-level compartment models, which categorize host’s epidemiological status (e.g., susceptible, exposed, infected, or recovered) and transition^1–3^. A potential drawback of these models is that they abstract the role of the environment in pathogen transmission process, as these compartment models were originally designed to describe epidemiological status changes of the hosts. Currently, there is no consensus on how to characterize environmental transferring dynamics of pathogens. Nevertheless, the environment plays a critical part in infectious disease dynamics since many pathogens (e.g., avian influenza virus, zoonotic *Escherichia coli* and *Salmonella spp*.) are transmitted partially or exclusively through contaminated environments^4–8^. Healthcare-associated infections (HAI, also known as nosocomial infections) are also facilitated by contacting surfaces and medical devices contaminated with pathogens, such as pathogenic *Clostridium difficile*^9,10^, Vancomycin-resistant *Enterococci* (VRE)^11,12^, and Methicillin-resistant *Staphylococcus aureus* (MRSA)^13,14^, and cause a tremendous amount of health and economic burden for society. Therefore, various novel modeling techniques have been developed and discussed to highlight the role of environment in infectious disease transmission especially HAIs^15–17^.

Metapopulation models are a type of spatial model which investigate interactions and movements among different subpopulations of (usually) the same species across time and space^18–23^. It is an extension of more conventional population-level compartment models that typically assume homogeneous mixing and implicit interactions within a population. Since pathogens transfer between heterogeneous hosts and environment, the metapopulation model could be extended to serve as a more appropriate modeling framework for capturing the explicit dynamics of pathogen movement, replication, and decay. Unlike more detailed agent-based models, which completely rely on simulation and do not have an explicit analytical form^1,17^, metapopulation models remain mathematically concise—usually expressed as an array of ordinary differential equations—and may be solved analytically without intensive computation.

Currently, most applications of epidemiological metapopulation models are restricted to direct host-to-host transmissions, focused exclusively on the host^20,24–26^. Pathogens are considered implicitly in these metapopulation models (usually via direct host-host contact) and rarely quantified (only population sizes of host in different epidemiological states are tracked). Nevertheless, host, pathogen, and environment together form the inseparable epidemiological triad, the fundamental concept and cornerstone of modern epidemiology. Thus, mathematical models that consider pathogens explicitly could potentially better characterize the system.

We propose extending metapopulation models for environmentally transmitted pathogens and shifting our perspective to model pathogen (sub)population dynamics, including pathogen movement, replication, and decay^27^. In our proposed modeling framework, both environment and host are considered as various “patches” or “dwellings” for pathogens, a recently proposed novel concept^28,29^. Pathogen population sizes are approximated using colony forming units (CFUs) for bacteria and fungi. The frequency and quantity of transferred pathogens are determined by host-environment contacts. Furthermore, other important components of the metapopulation model, including microbe spatial structure, growth, motility, local carrying capacity, and sociobiology, have been already studied and discussed in the existing literature^30–35^.

We demonstrate metapopulation model as a theoretically sound and pragmatic approach for epidemiologic analyses at the pathogen level. In particular, we show its application for simulating and analyzing pathogen transferring dynamics, including system equilibrium characteristics, uni-directional transferring, and rescue effects from source-sink dynamics. These concepts and approaches are critical for developing and evaluating more effective control strategies for HIA and improving patient safety^12,36–38^.

The objectives of this study are:

1. Develop a generalized metapopulation modeling framework to track pathogen transferring in a semi-closed healthcare system, and;
2. Quantify pathogen circulation between environment and hosts and evaluate potential controlling strategies in three case studies (pure transferring dynamics, source-sink dynamics, and external disinfection).

## Methods

### The Generalized Metapopulation Model for Pathogen Transferring

We first construct a generalized metapopulation model to characterize and quantify HAI pathogen transferring and circulation between the environment and a group of heterogeneous hosts that contact the environment in a semi-closed healthcare setting. We consider the environmental transferring of pathogens and exclude direct host-to-host contact. We will later discuss extensions of this modeling framework for direct transmission pathway. The pathogen metapopulation consists of all the pathogen subpopulations in each of the hosts and environments. For ease of exposition, we consider only one homogeneous environment; however, this modeling framework easily accommodates multiple heterogeneous environments.

From metapopulation perspective, population dynamics of each pathogen subpopulation can be formulated as the following discrete-time difference equations in system 1:

1a $${X}_{t}={X}_{t-1}+{\rm{\Delta }}{X}_{t}$$

1b $${X}_{t}={X}_{t-1}+{X}_{{\rm{inflow}}}-{X}_{{\rm{outflow}}}+{X}_{{\rm{birth}}}-{X}_{{\rm{death}}}$$

where *X*_*t*_ denotes the amount of pathogens (population size, could be approximated by CFUs) in a given subpopulation at time *t*, which depends on population size *X*_*t*−1_ and change in population size Δ*X*_*t*_, consisting of a total of four possible terms: total pathogen inflow *X*_inflow_ (e.g., transferred from all hosts to the environment, using environment subpopulation as example); total pathogen outflow *X*_outflow_ (e.g., transferred from the environment to all hosts); pathogen birth *X*_birth_ (e.g., replicated within the environment between *t* − 1 and *t*); and pathogen death *X*_death_ (e.g., decayed in the environment between *t* − 1 and *t*). The two terms increasing the subpopulation size have positive signs (i.e., inflow and birth) while the two terms decreasing population size have negative signs (i.e., outflow and death).

**Figure 1:**
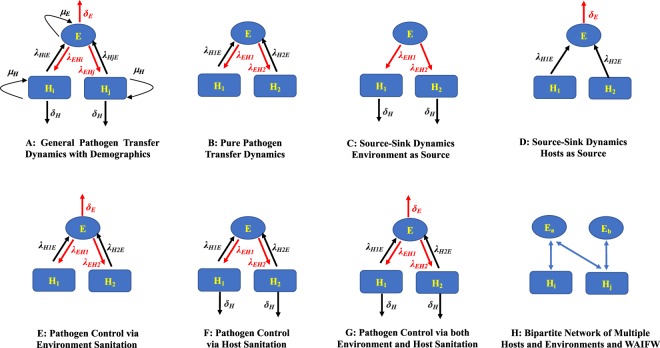
Model Diagrams of General Pathogen Transferring Dynamics, Pure Pathogen Transferring Dynamics, Source-Sink Dynamics, and Pathogen Controlling Dynamics. Note: *E*: one homogeneous environment; Hi: host i; μH: pathogen replicate rate in host; δH: pathogen death rate in host; *λ*: pathogen transferring rate; *μ*_*E*_: pathogen replicate rate in environment; *δ*_*E*_: pathogen death rate in environment; WAIFW: Who-Acquire-Infection-From-Whom; red color indicates pathogen flow out of environment (*E*).

A generalized, continuous-time metapopulation model for pathogen population dynamics can be formulated as a system of ordinary differential equations (ODEs) deriving from system 1. Let *E* and *H*_*i*_ denote the instantaneous amounts of pathogens in the environment and host *i* ∈ {1, …, *N*}, where *N* is the number of hosts. Then, our generalized model for environmental pathogen transferring is expressed as the following ODE system 2:

2a $$\frac{dE}{dt}=\sum _{i=1}^{N}{\lambda }_{{H}_{i}E}{H}_{i}-\sum _{i=1}^{N}{\lambda }_{E{H}_{i}}E+{\mu }_{E}E-{\delta }_{E}E,$$

2b $$\frac{d{H}_{i}}{dt}={\lambda }_{E{H}_{i}}E-{\lambda }_{{H}_{i}E}{H}_{i}+{\mu }_{{H}_{i}}{H}_{i}-{\delta }_{{H}_{i}}{H}_{i},\,\forall i\in \{1,\ldots ,N\}$$

where, for all *i* ∈ {1, …, *N*}, parameters ${\lambda }_{{H}_{i}E}$ and ${\lambda }_{E{H}_{i}}$ denote pathogen transferring rates from host *H*_*i*_ to environment *E* and *vice versa*, respectively; parameters *μ*_*E*_ and ${\mu }_{{H}_{i}}$ denote pathogen replication rates within environment *E* and host *H*_*i*_, respectively, and parameters *δ*_*E*_ and ${\delta }_{{H}_{i}}$ denote pathogen decay rates in environment *E* and host *H*_*i*_, respectively. We focus on pathogen transferring instead of transmission. The transferring terms are considered linear and associated with either host or environment. This is different from host-level compartment models (e.g., SIR-type models) where transmission terms are generally nonlinear^1^. This generalized model is illustrated in Fig. 1A with *N* = 2 hosts as an example.

Pathogen transferring rates ${\lambda }_{{H}_{i}E}$ and ${\lambda }_{E{H}_{i}}$ can be expressed as the product of two factors: (1) the contact frequency between host and environment (which can be measured and quantified using proximity loggers or radio frequency ID tags^39–41^), and (2) the likelihood of pathogens transferring between host and environment. Because the two uni-directional transferring events are mutually exclusive and collectively exhaustive, the two pathogen transferring probabilities sum to unity. For notational brevity and introduction to this modeling framework, we use transferring rates ${\lambda }_{{H}_{i}E}$ and ${\lambda }_{E{H}_{i}}$, and do not further divide them into more complicated interactions.

### Case Study 1: Pure Pathogen Transferring Model

The pure transferring model is obtained from the generalized model where pathogen birth (replication) and death (decay) terms are omitted. Such models are valid since many HAI pathogens replicate and decay in negligible amounts within relatively short periods of time. In these studies, the system becomes a closed system where pathogens only circulate among environment and hosts. This case study is illustrated in Fig. 1B for *N* = 2 hosts, and its corresponding ODE system is shown as follows in system 3:

3a $$\frac{dE}{dt}=\sum _{i=1}^{N}{\lambda }_{{H}_{i}E}{H}_{i}-\sum _{i=1}^{N}{\lambda }_{E{H}_{i}}E,$$

3b $$\frac{d{H}_{i}}{dt}={\lambda }_{E{H}_{i}}E-{\lambda }_{{H}_{i}E}{H}_{i},\,\forall i\in \{1,\ldots ,N\}$$

We derive the analytical solution to the system equilibrium, evaluate the system’s long-term stability of the equilibrium, and provide a numerical simulation demonstrating pathogen transferring dynamics for this system.

### Case Study 2: Source-Sink Dynamics Model for Pathogen Control

Source-sink dynamics model is a special case of the generalized metapopulation model, where its pathogen transferring is uni-directional (i.e., from source to sink). In these models, pathogens originate from the source and transfer to the sink, where pathogen deaths subsequently occur. Two types of source-sink dynamics models are developed where either environment or hosts plays the role of source (consequently, the other serves as sink). Source-sink dynamics where hosts as source is depicted in Fig. 1C (for *N* = 2 hosts, assuming pathogen decay rates are the same across all hosts, i.e., ${\delta }_{{H}_{i}}\equiv {\delta }_{H}$) and the ODE system is expressed as system 4:

4a $$\frac{dE}{dt}=-\,\sum _{i=1}^{N}{\lambda }_{E{H}_{i}}E,$$

4b $$\frac{d{H}_{i}}{dt}={\lambda }_{E{H}_{i}}E-{\delta }_{H}{H}_{i},\,\forall i\in \{1,\ldots ,N\}.$$

Source-sink dynamics for the other type (environment as source) is depicted in Fig. 1D (for *N* = 2 hosts), and the ODE system is expressed in system 5:

5a $$\frac{dE}{dt}=\sum _{i=1}^{N}{\lambda }_{{H}_{i}E}{H}_{i}-{\delta }_{E}E,$$

5b $$\frac{d{H}_{i}}{dt}=-\,{\lambda }_{{H}_{i}E}{H}_{i},\,\forall i\in \{1,\ldots ,N\}.\,$$

We derive analytical solutions to system equilibria and provide numerical simulations demonstrating pathogen transferring dynamics for these two source-sink dynamics models.

### Case Study 3: Pathogen Control via Disinfection

Pathogen control via disinfection model is an extension of the pure transferring model where pathogens are removed from the environment and/or hosts via disinfection/sanitation. Here, we consider more realistic settings and incorporate external forces to reduce pathogen population size either in environment or in hosts, while still maintaining bi-directional pathogen transferring. Three types of pathogen control via disinfection models can be derived where: (1) only environment is disinfected; (2) only hosts are sanitized, and; (3) both environment and hosts are disinfected/sanitized. We provide the models for these types below and compare these three different pathogen controlling strategies using numeric simulations.

For controlling pathogens in the environment, we consider a janitor who comes in regularly at a given arrival rate to apply disinfectants in the healthcare facility (typically once or twice a day, also considered as disinfectant application rate). We assume that the janitor neither sheds pathogens to environment nor acquires pathogens from environment, and the disinfection probability is a fixed value (i.e., probability of killing the pathogen; usually specified by disinfectant manufacturer). We use a single parameter *δ*_*E*_ for the combined effect of disinfectant application rate and disinfection probability. This case study is illustrated in Fig. 1E for *N* = 2 hosts, and its corresponding ODE system is shown in system 6:

6a $$\frac{dE}{dt}=\sum _{i=1}^{N}{\lambda }_{{H}_{i}E}{H}_{i}-\sum _{i=1}^{N}{\lambda }_{E{H}_{i}}E-{\delta }_{E}E,$$

6b $$\frac{d{H}_{i}}{dt}={\lambda }_{E{H}_{i}}E-{\lambda }_{{H}_{i}E}{H}_{i},\,\forall i\in \{1,\ldots ,N\},$$

For controlling pathogens in hosts, we model hosts who regularly perform self-sanitation (e.g., washing hands or using hand sanitizer) at a given rate. Similar to the first type of environment sanitation, we use parameter *δ*_*H*_ for the sanitation rate of hosts. This case study is illustrated in Fig. 1F for *N* = 2 hosts, and its corresponding ODE system is shown in system 7:

7a $$\frac{dE}{dt}=\sum _{i=1}^{N}{\lambda }_{{H}_{i}E}{H}_{i}-\,\sum _{i=1}^{N}{\lambda }_{E{H}_{i}}E$$

7b $$\frac{d{H}_{i}}{dt}={\lambda }_{E{H}_{i}}E-{\lambda }_{{H}_{i}E}{H}_{i}-{\delta }_{H}{H}_{i},\,\forall i\in \{1,\ldots ,N\},$$

For controlling pathogens in both environment and hosts, we consider both disinfection and sanitation that influence pathogen transferring dynamics. This case study is illustrated in Fig. 1F for *N* = 2 hosts, and its corresponding ODE system is shown in system 8:

8a $$\frac{dE}{dt}=\sum _{i=1}^{N}{\lambda }_{{H}_{i}E}{H}_{i}-\sum _{i=1}^{N}{\lambda }_{E{H}_{i}}E-{\delta }_{E}E,$$

8b $$\frac{d{H}_{i}}{dt}={\lambda }_{E{H}_{i}}E-{\lambda }_{{H}_{i}E}{H}_{i}-{\delta }_{H}{H}_{i},\,\forall i\in \{1,\ldots ,N\},$$

Since this is the most comprehensive model for HAI control in this study, we also evaluate the relative importance of the parameters (${\lambda }_{E{H}_{i}}$, ${\lambda }_{{H}_{i}E}$, *δ*_*E*_, *δ*_*H*_), and their associated processes; for example, *δ* is associated with disinfection and sanitation through sensitivity analysis using Latin hypercube sampling^42^. Pearson partial rank correlation coefficients (PRCC) on the maximum amount of pathogen in both hosts combined at time *t* are calculated. Note that we are not using the sum of maximum pathogen on either host, as the time of maximum pathogen occurrence could be different between two hosts. Additionally, it is difficult to evaluate the potential risk of HAI through time. As shown later in the results, this dynamic system has trivial equilibrium as the only system equilibrium, and such equilibrium will not be an effective measurement to evaluate parameter sensitivity. In general, parameters with large absolute values and corresponding small *p*-values are considered more influential in the model. A complete description of parameters and their values used in simulation is provided in Table 1.

**Table 1:** Model Parameter Description, Values, and PRCC Result.

Parameter	Description	Value	Range	PRCC	*p*-value
*λ*_*EH1*_	Pathogen transfer rate from environment to host 1	0.2	[0.05–0.5]	0.17	0.12
*λ*_*EH2*_	Pathogen transfer rate from environment to host 2	0.3	[0.05–0.5]	0.17	0.12
*λ*_*H1E*_	Pathogen transfer rate from host 1 to environment	0.4	[0.05–0.5]	−0.24	0.08
*λ*_*H2E*_	Pathogen transfer rate from host 2 to environment	0.5	[0.05–0.5]	−0.24	0.08
*δ*_*E*_	Disinfection rate in environment	0.1	[0.05–0.25]	−0.39	0.03*
*δ*_*H*_	Disinfection rate in host	0.2	[0.05–0.25]	−0.55	<0.01**

Note: PRCC (partial rank correlation coefficient) is used to assess parameter sensitivity and relative importance for the target variable (maximum pathogen on both hosts in this study). A positive PRCC value indicates positive correlation with target variable and negative value indicates negative correlation. A larger absolute PRCC value (closer to 1) corresponds to higher parameter sensitivity. Simulations are based on the same initial condition ${E}^{0}=50,\,{H}_{1}^{0}=30,\,{H}_{2}^{0}=30$. Parameter values are hypothetical and for demonstrating the feasibility of the modeling framework.

## Results

### Case Study 1: How Pathogen Circulate and Stabilize in System

In this pure transferring case (corresponding to system 3), pathogens never leave the system nor do new pathogens enter. Therefore, the dynamic equilibrium (steady-state) of this system can be achieved and the equilibrium of pathogen distribution in environment and each host can be derived analytically. We demonstrate that system equilibrium depends on the specific parameterization (i.e., numeric values of *λ*s). Because this is a closed system, we scale *E* and *H*_*i*_ as follows (*E*′ and ${{H}_{i}}^{^{\prime} }$ as percentages) in system 9:

9a $$E^{\prime} =\frac{E}{E+{\sum }_{i=1}^{N}{H}_{i}},$$

9b $${H}_{i}^{^{\prime} }=\frac{{H}_{i}}{E+{\sum }_{j=1}^{N}{H}_{j}},\,\forall i\in \{1,\ldots ,N\},$$

Note that the right-hand side of system 3 for this case study is a singular matrix. Since we have scaled *E* and *H*_*i*_, an additional equation ${\bar{E}}^{{\rm{^{\prime} }}}+\sum _{i=1}^{N}{\bar{H}}_{i}^{{\rm{^{\prime} }}}=1$ is added to derive the system equilibrium for this closed system ($\bar{E}^{\prime}$ and ${\bar{H}}_{i}^{{\rm{^{\prime} }}}$ represent scaled pathogen distribution at equilibrium in environment *E* and host *H*_*i*_, respectively). For the case that we have proposed (one environment and two hosts), the equilibrium for scaled pathogen distribution in environment and each host is shown as follows in system 10:

10a $$\bar{E}^{\prime} =\frac{{\lambda }_{{H}_{1}E}{\lambda }_{{H}_{2}E}}{{\lambda }_{E{H}_{1}}{\lambda }_{{H}_{2}E}+{\lambda }_{{H}_{1}E}{\lambda }_{{H}_{2}E}+{\lambda }_{E{H}_{2}}{\lambda }_{{H}_{1}E}},$$

10b $${\bar{H}}_{1}^{{\rm{^{\prime} }}}=\frac{{\lambda }_{E{H}_{1}}{\lambda }_{{H}_{2}E}}{{\lambda }_{E{H}_{1}}{\lambda }_{{H}_{2}E}+{\lambda }_{{H}_{1}E}{\lambda }_{{H}_{2}E}+{\lambda }_{E{H}_{2}}{\lambda }_{{H}_{1}E}},$$

10c $${\bar{H}}_{2}^{{\rm{^{\prime} }}}=\frac{{\lambda }_{E{H}_{2}}{\lambda }_{{H}_{1}E}}{{\lambda }_{E{H}_{1}}{\lambda }_{{H}_{2}E}+{\lambda }_{{H}_{1}E}{\lambda }_{{H}_{2}E}+{\lambda }_{E{H}_{2}}{\lambda }_{{H}_{1}E}},$$

We also demonstrate that for the more general scenario (one environment and *N* hosts), the system equilibria of pathogen distributions can be formulated as system 11:

11a $$\bar{E}^{\prime} =\frac{{\prod }_{i=1}^{N}{\lambda }_{{H}_{i}E}}{{\prod }_{i=1}^{N}{\lambda }_{{H}_{i}E}+{\sum }_{i=1}^{N}{\lambda }_{E{H}_{i}}{\prod }_{j=1}^{N,\,j\ne i}{\lambda }_{{H}_{j}E}},$$

11b $${\bar{H}}_{i}^{{\rm{^{\prime} }}}=\frac{{\lambda }_{E{H}_{i}}{\prod }_{j=1}^{N,\,j\ne i}{\lambda }_{{H}_{j}E}}{{\prod }_{i=1}^{N}{\lambda }_{{H}_{i}E}+{\sum }_{i=1}^{N}{\lambda }_{E{H}_{i}}{\prod }_{j=1}^{N,\,j\ne i}{\lambda }_{{H}_{j}E}},\,{\rm{\forall }}i\in \{1,\ldots ,N\}$$

These equilibria (system 10 and 11) are governed by specific system parameterizations (i.e., depending on *λs*, independent of initial pathogen distributions in hosts and environment) and determine how pathogens distribute in environment and different hosts in long-run when system reaches equilibrium. These equilibria can be used as practical guidance to identify potential “high-risk” environment/hosts (i.e., having a relatively large value of equilibrium; for example, any environment and/or host having more than $\frac{1}{1+N}$ of total pathogen could be considered as “high-risk”, where $\frac{1}{1+N}$ represents homogeneous pathogen distribution across all hosts and environment). Empirical studies to identify “high-risk” fomites in healthcare facilities are generally based on contact structure of fomites (i.e., contact duration and frequency between host and fomite^9,10,12^). Our findings from pathogens perspective could help develop more specific controlling strategies against HAI. Another important implication is that the system equilibrium does not rely on initial condition (i.e., initial distribution of pathogens in the system), as the system will eventually reach its equilibrium, which could be readily derived analytically in system 10 and more generally system 11.

**Figure 2:**
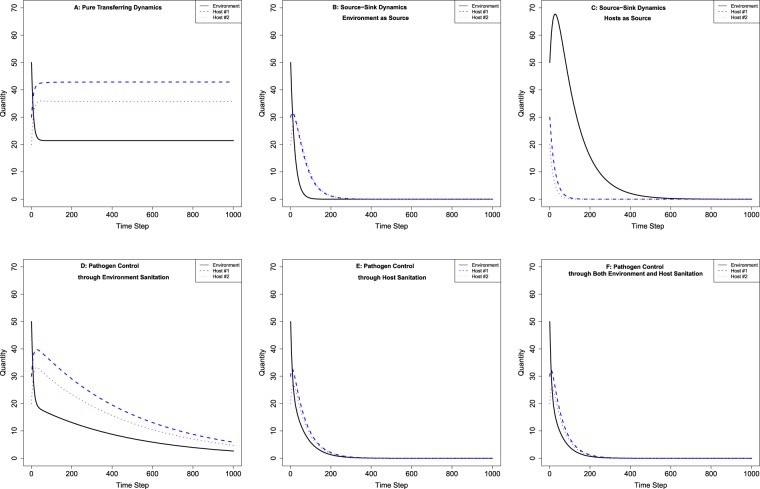
Numerical Results of Case Studies of Pure Transfer Dynamics, Source-Sink Dynamics, and Pathogen Control Dynamics Models. Note: Panel (A) pure transferring dynamics; (**B**) for source-sink dynamics where environment as source; (**C**) for source-sink dynamics where hosts as source; (**D**) for pathogen control dynamics through environment sanitation; (**E**) for pathogen control dynamics through host sanitation; (**F**) for pathogen control dynamics through both environment and host sanitation. Note that pure transferring dynamics model has non-trivial system equilibrium, and all the other cases have trivial equilibrium (i.e., no pathogen eventually exists in the system).

In this case, we do not consider the trivial solution to the system equilibrium ${\bar{E}}^{{\rm{^{\prime} }}}={\bar{H}}_{1}^{{\rm{^{\prime} }}}={\bar{H}}_{2}^{{\rm{^{\prime} }}}=0$ because the parameter values (transfer rates) should always be nonzero. Nevertheless, in the next case study we demonstrate this trivial solution to the system equilibrium is important and meaningful. Besides the analytical solution of the system equilibrium that we have demonstrated, we also provide the numeric simulation of this system, with parameter set

$${\lambda }_{E{H}_{1}}=0.2,\,{\lambda }_{E{H}_{2}}=0.3,\,{\lambda }_{{H}_{1}E}=0.4,\,{\lambda }_{{H}_{2}E}=0.5$$

and initial condition of pathogen amount ${E}^{0}=50,\,{H}_{1}^{0}=30,\,{H}_{2}^{0}=30$ as an example. The population dynamics of subpopulations in each of the two hosts and environment is shown in Fig. 2A, and we confirm that the system converges to its equilibrium (${\bar{E}}^{{\rm{^{\prime} }}}=21,\,{\bar{H}}_{1}^{{\rm{^{\prime} }}}=43,\,{\bar{H}}_{2}^{{\rm{^{\prime} }}}=36$), which could be derived from system 10. In this example, host H_1_ has the largest amount of pathogen on it.

### Case Study 2: Why Trivial Solution of System Equilibrium is Actually Critical

In the source-sink dynamics models, the systems are not closed and trivial solution ${\bar{E}}^{{\rm{^{\prime} }}}={\bar{H}}_{1}^{{\rm{^{\prime} }}}={\bar{H}}_{2}^{{\rm{^{\prime} }}}=0$ is the only solution to the system equilibrium (no matter whether environment or hosts serve as source or sink, this can be solved by setting the right-hand side of the equations in systems 4 and 5 as zero). Furthermore, this system has stable equilibrium (more generally, it is shown that for such source-sink system with *N* hosts and *M* environments, ${\bar{E}}_{i}^{{\rm{^{\prime} }}}={\bar{H}}_{j}^{{\rm{^{\prime} }}}=0,$ $\forall i\in \{1,\ldots ,M\},\,\forall j\in \{1,\ldots ,N\}$, equilibrium is stable). Mathematically speaking, a trivial solution is generally not very meaningful, but from a practical perspective, such a trivial solution actually represents the ultimate goal for HAI control: to completely remove pathogens from the system. Since all terms in this model are linear, we can combine potential pathogen replication (not originally considered) with pathogen outflow terms and formulate the “effective” decay rate in either environment and hosts. We show that as long as this effective decay rate remains positive (i.e., pathogen birth rate is smaller than decay rate), trivial solution of system equilibrium will also be valid, hence all pathogens will eventually be eradicated from the system.

We show numeric simulations to two types of source-sink dynamics models, the first being environment as source (system 4), with hypothetical parameter set ${\lambda }_{E{H}_{1}}=0.2,\,{\lambda }_{E{H}_{2}}=0.3,\,{\delta }_{H}=0.1$and the same initial condition as case study 1: ${E}^{0}=50,\,{H}_{1}^{0}=30,\,{H}_{2}^{0}=30$. Illustration of system dynamics is provided in Fig. 2B, where pathogen population size in environment keeps declining to zero monotonically and population sizes in both hosts raise first and then both decay to zero. These numeric results demonstrate that trivial solution to system equilibrium $\bar{E}^{\prime} ={\bar{H}}_{1}^{^{\prime} }={\bar{H}}_{2}^{^{\prime} }=0$ is indeed achieved.

In the second simulation, hosts are sources (system 5), and we set hypothetical parameters as ${\lambda }_{{H}_{1}E}=0.4,$ ${\lambda }_{{H}_{2}E}=0.5,\,{\delta }_{E}=0.2$, keeping the same initial condition ${E}^{0}=50,\,{H}_{1}^{0}=30,\,{H}_{2}^{0}=30$. The numeric solution of population dynamics is provided in Fig. 2C, where pathogen population sizes in both hosts decrease to zero monotonically, and population size in environment raise initially, but then decrease to zero as well. These numeric results also show that trivial solution $\bar{E}^{\prime} ={\bar{H}}_{1}^{^{\prime} }={\bar{H}}_{2}^{^{\prime} }=0$ (more generally, ${\bar{E}}_{i}^{^{\prime} }={\bar{H}}_{j}^{^{\prime} }=0,\,\forall \,i\in \{1,\ldots ,M\},$ $\forall \,j\in \{1,\ldots ,N\}$) is the stable equilibrium of this system.

Both analytical and numerical solutions to system equilibrium in these two types of source-sink dynamics model demonstrate that pathogen populations can be eradicated from the system. However, the true population dynamics in these two types of models differ. Although both conditions (cutting off host-environment transferring pathway versus cutting off environment-host transferring pathway) lead to total eradication of pathogens in the system, their efficacy and efficiency (i.e., convergence time to equilibrium) differ. When environment is the source, pathogen population sizes actually increases in hosts initially (hosts only receive pathogen from environment and never shed pathogen back), which could lead to potential HAIs (if we further consider the within-host infection dynamics). This provides a feasible solution to explicitly quantify, carefully evaluate, and accurately compare different HAI controlling efforts, and also develop more effective controlling strategies. We can further evaluate potential economic cost (constraints) to achieve the goal of HAI control by shutting down a specific pathogen transferring pathway, discussed in detail in discussion section of this paper.

### Case Study 3: Evaluating Efforts for HAI Control through Disinfection

While it might not be feasible to completely cut off either pathogen transferring pathway, a more practical solution is to control pathogen population in the environment, in hosts, or in both, via external disinfection and sanitation.

Similar to source-sink dynamics model, we demonstrate that the trivial solution to system equilibrium $\bar{E}^{\prime} ={\bar{H}}_{1}^{^{\prime} }={\bar{H}}_{2}^{^{\prime} }=0$ (and more generally ${\bar{E}}_{i}^{{\rm{^{\prime} }}}={\bar{H}}_{j}^{{\rm{^{\prime} }}}=0,\,{\rm{\forall }}\,i\in \{1,\ldots ,M\},\,{\rm{\forall }}\,j\in \{1,\ldots ,N\}$) is the only solution, regardless of whether the controlling effort is applied to environment and/or hosts. There are extra outflow terms in addition to the previously discussed closed system 3 (corresponding to case study 1, pure transferring system), so eventually the systems in case study 3 (systems 6–8) will reach the trivial solution of system equilibrium. Thus, from HAI controlling perspective, it is not a matter of “if”, a more practical question would be “when” we expect pathogens to be thoroughly eradicated from the system. We demonstrate three sets of numeric simulations corresponding to HIA controlling strategies, focusing on environment, hosts, and both, respectively (systems 6–8). Further, pathogen replication could also be considered and combined within the “effective” controlling rate (i.e., disinfection rate minus replication rate). We demonstrate that as long as this effective controlling rate remains positive, trivial solution of system equilibrium will still be the only solution, though the rate of convergence will be different and depends on the new effective controlling rate.

Hypothetical parameter settings have the same pathogen transferring parameters as pure pathogen transferring case for consistency: ${\lambda }_{E{H}_{1}}=0.2,\,{\lambda }_{E{H}_{2}}=0.3,\,{\lambda }_{{H}_{1}E}=0.4,\,{\lambda }_{{H}_{2}E}=0.5$, except differing in pathogen controlling parameters *δ*_*E*_ = 0.1, *δ*_*H*_ = 0; *δ*_*E*_ = 0, *δ*_*H*_ = 0.2; and *δ*_*E*_ = 0.1,*δ*_*H*_ = 0.2 for these three simulations, respectively. These parameter values are chosen to reflect different rates in disinfection: usually host sanitation (e.g., hand washing) is more frequent than environment sanitation (e.g., disinfection by a janitor). All three simulations start with the same initial condition: ${E}^{0}=50,\,{H}_{1}^{0}=30,\,{H}_{2}^{0}=30$. Pathogen population dynamics are shown in Fig. 2E through Fig. 2G for the three respective controlling strategies. Although pathogens decline and converge to system equilibrium (0) in all three simulations, the rates of convergence to system equilibria are different.

**Figure 3:**
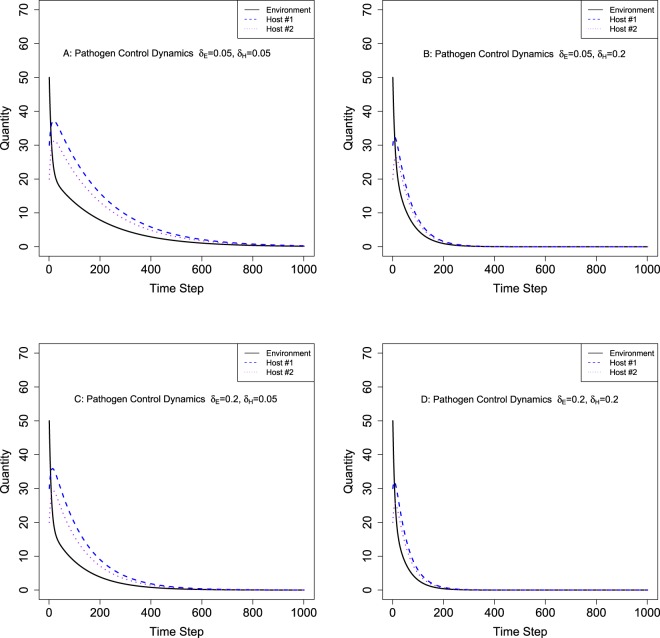
Numerical Results of Pathogen Control Dynamics Models with Varying Control Parameters (δ_E_, δ_H_). Note: Pathogen control parameter *δ*_*E*_ is associated with environment sanitation and *δ*_*H*_ is associated with host sanitation. 3A through 3D have varying hypothetical parameters.

**Figure 4:**
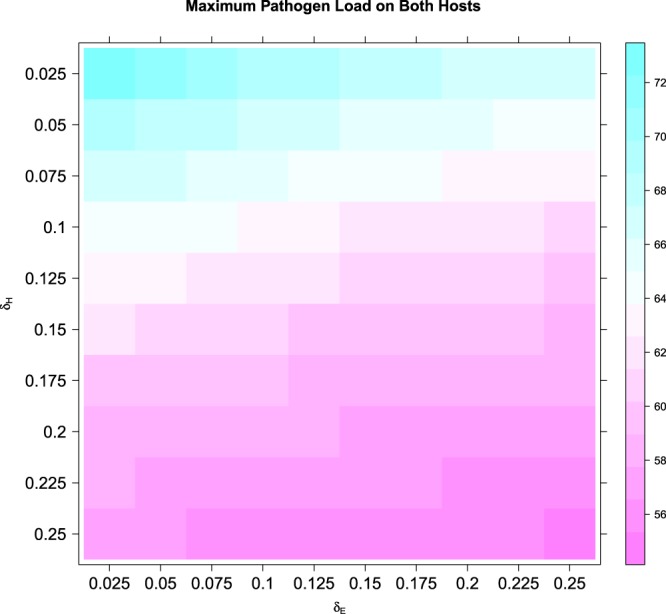
Maximum Pathogen Load on Both Hosts with Varying Control Parameters (δ_E_, δ_H_). Note: Pathogen control parameter *δ*_*E*_ is associated with environment sanitation and *δ*_*H*_ is associated with host sanitation. Maximum pathogen load is calculated as the maximum value of pathogen population size on both hosts combined at any given time during simulation. The parameters are hypothetical.

We also quantify model parameters’ relative importance based on partial rank correlation coefficient (PRCC). The results are provided in Table 1. Parameters corresponding to pathogen circulation (transferring parameters *λ*s) are considered less important (smaller absolute PRCC values) than parameters associated with pathogen controlling (*δ*_*E*_ and *δ*_*H*_). Furthermore, host sanitation parameter *δ*_*H*_ is more sensitive (i.e., it changes system dynamics more substantially given the same amount of parameter value change) than environment disinfection parameter *δ*_*E*_. These findings imply that increasing pathogen controlling efforts (e.g., increasing disinfection rates in the system, especially on hosts) could lead to faster eradication of pathogens in the system and used as useful guidance to reduce HAI. Additional numeric simulation results based on combinations of different *δ*_*E*_ and *δ*_*H*_ values are provided in Fig. 3A through Fig. 3D to illustrate the influence of these parameters on pathogen population dynamics and potential effects of HAI controlling. Maximum numbers of total pathogens (i.e., maximum burden of pathogens) on both hosts with varying pathogen controlling parameters (*δ*_*E*_ and *δ*_*H*_) are illustrated in Fig. 4.

## Discussion

In this study, we have developed a versatile metapopulation modeling framework from pathogen’s perspective and demonstrate how this model can investigate pathogen transferring and circulation in a closed or semi-closed system, such as healthcare settings. Results show that for a closed system (pure pathogen transferring case study), equilibrium distribution of pathogens can be achieved analytically. These results regarding long-term equilibrium of pathogen distributions on hosts and environment serve as potential guidance to identify and evaluate high-risk areas (also known as fomites) and/or personnel in HAI. For a semi-closed system (e.g., applying pathogen controlling), we are able to achieve the goal of pathogen eradication and different controlling strategies (via host sanitation, environment sanitation, or both, with differing efficacy), which could be quantified by time until equilibrium distribution. The relatively simple model structure (e.g., all linear transferring, replication, decay, and controlling terms, instead the of more complicated nonlinear transmission terms commonly utilized in host-level compartment models) enables the easy use of this model, explicit interpretation of the results, and straightforward implications of pathogen controlling for researchers (especially in the field of HAI), even without intensive training in mathematical modeling. Future study directions include investigating specific HAI pathogen transferring dynamics such as MRSA, VRE, and *C. difficile*. Further cost-effectiveness of these different methods can be evaluated by adding corresponding financial constraints (e.g., cost of disinfectant, labor, and cost associated with treating HAIs, etc). Other applications include exploring macroparasites whose population size could be more explicitly measured^43^. Both macroparasites and pathogens share similar population dynamics characteristics (e.g., replication, decay, inflow, and outflow), hence they can be analyzed with our modeling framework as well.

The novelty and uniqueness of our study is that our modeling framework is from pathogen’s perspective. Even for pathogens that cannot be directly quantified (e.g., viruses), occupancy metapopulation model^19^ can be applied to reflect whether or not the patch (either host or environment) is occupied by the pathogen (though the actual quantity of pathogen is not available), as there is a direct link between the occupancy metapopulation model and more conventional host-level compartment model. The 0–1 occupancy metapopulation model is in fact another representation of the SIS-type compartment model, where 0 corresponds to susceptible hosts who are not infected with the pathogen, and 1 corresponds to infected hosts with the pathogen. Once the pathogen leaves the host (either moved or removed via disinfection), the infected host comes back to susceptible state.

Since pathogen, host, and environment collectively form the inseparable epidemiological triad, this modeling framework is, indeed, an individual-based model from host’s perspective (even for the 0–1 occupancy metapopulation model, as we are still tracking pathogens among individual hosts). For instance, pathogen transferring is determined by host-environment contact structure, hence the pathogen transferring rates corresponds to the individual host’s contact rate with the environment, as well as the janitor’s disinfecting schedule. With recent technological advances, it is relatively easy to capture and characterize host-environment contact structure with high accuracy and precision. Recent advances include radio frequency ID tags, proximity loggers, and, even more traditionally, close circuit TVs coupled with advanced image-processing and artificial intelligence algorithms^39–41^. In the case studies discussed in this paper, we set pathogen controlling parameter *δ* consistent among multiple hosts, but it can be easily tweaked to characterize individual host’s different behavior (e.g., hand-sanitation frequency). Additionally, the individual host’s demographic and clinical characteristics, such as susceptibility to certain pathogen, can be incorporated to further model within-host infection dynamics^44,45^. For instance, risk factors such as age, gender, and ethnicity group can be used to quantify or adjust susceptibility. Based on the results from our modeling framework (i.e., maximum amount of pathogen in hosts at time *t*), we can evaluate the real-time infection risk for each individual host in the system. Moreover, this versatile modeling framework can handle variable host population size through time (number of hosts *N* is not fixed, e.g., representing a healthcare setting with high fluidity of patient flow^17^). Once between-host transfer dynamics and within-host infection dynamics are coupled, we can infer host’s epidemiological state and quantify potential pathogen shedding to further parameterize the pathogen transfer rate from host to environment *λ*_*HE*_.

Although we initially refrain this modeling framework to pathogen transferring between host and environment, other transmission pathways such as direct host-host transmission can also be included. As discussed earlier, environments and hosts are both patches from pathogens perspective, and the host-host pathogen transferring pathway is a similar process, provided that we can quantify host-host contact structure. This can be further investigated by network analysis for hosts’ contact structure. For a closed or semi-closed system such as healthcare settings, measuring and characterizing host-host contact network with high accuracy is technically feasible. Important epidemiological metrics for infectious diseases such as basic reproduction number (*R*_0_) could be derived as long as host’s epidemiological states are determined. Furthermore, even if direct host-host contact structure cannot be characterized, we are also able to infer the “who-acquire-infection-from-whom” (WAIFW) information using this modeling framework and track pathogen flow among different host species^46–48^. Note that though this modeling framework can accommodate multiple environments and various hosts, it is possible to track how pathogen flows between different environments and hosts. In this case, we use the bipartite network where environments are represented as level-1 vertices and hosts are level-2 vertices, and pathogen flow can be traced to infer the WAIFW information (see Fig. 1).

Regardless of the affability of our proposed metapopulation modeling framework, it is best suited for closed/semi-closed system (such as healthcare settings), where detailed contact structure and other parameters (such as disinfection rates) can be measured and quantified. Nevertheless, the beauty of metapopulation model lies in its scalability: from individual to population and to metapopulation levels. Similarly, we can treat a single ward within a hospital, or even an entire healthcare facility, as a large metapopulation for pathogens, as patients proceed within or among hospitals, creating the potential pathogen flow. With modern information technology such as electronic medical record (EMR) and/or electronic health record (EHR), we are able to track patient flow explicitly and build more effective early warning systems for potential HAIs among multiple healthcare facilities^49^.
